# Electroreceptive and Mechanoreceptive Anatomical Specialisations in the Epaulette Shark (*Hemiscyllium ocellatum*)

**DOI:** 10.1371/journal.pone.0049857

**Published:** 2012-11-30

**Authors:** Marit Winther-Janson, Barbara E. Wueringer, Jamie E. Seymour

**Affiliations:** 1 School of Marine and Tropical Biology, James Cook University, Cairns, Queensland, Australia; 2 Centre for Biodiscovery and Molecular Development of Therapeutics, James Cook University, Cairns, Queensland, Australia; University of Alberta, Canada

## Abstract

The arrangement of the electroreceptive ampullary system and closely related mechanoreceptive lateral line canal system was investigated in the epaulette shark, *Hemiscyllium ocellatum*. The lateral line canals form an elaborate network across the head and are continuously punctuated by pores. Ampullary pores are distributed in eleven distinct pore fields, and associated ampullary bulbs are aggregated in five independent ampullary clusters on either side of the head. Pores are primarily concentrated around the mouth and across the snout of the animal. We provide the anatomical basis for future behavioural studies on electroreception and mechanoreception in epaulette sharks, as well as supporting evidence that the electroreceptive ampullary system is specialised to provide behaviourally relevant stimuli. In addition, we describe ampullary pores distributed as far posteriorly as the dorsal fin and thus reject the assumption that ampullary pores are restricted to the cephalic region in sharks.

## Introduction

How an organism relates to the physical world depends upon its sensory capabilities [1]–[2]. Marine elasmobranchs are equipped with olfactory, auditory, visual, mechanoreceptive, electroreceptive, touch and gustation sensory modalities [3]–[8]. The physical operating range of each sense is different and determines its ecological application [9]. For example, touch and gustation are close-range senses, while audition and olfaction function over large distances of several kilometres [9]. Specialised morphological adaptations within each sensory organ further dictate which stimuli can be perceived, thus defining the realm of a species' niche [10].

The mechanosensory lateral line system is an example of a close range sensory system which enables elasmobranchs to detect local water displacement. The distribution of the canals in the epidermis determines the receptive field of the functional units, namely the canal neuromasts [11]–[12]. These neuromasts are comprised of sensory and supportive cells bound by a gelatinous cupula [13]. As water movement creates viscous drag inside the lateral line canals the cupula is displaced, which in turn stimulates the associated nerve [13].

Interestingly, lateral line canals can be pored or non-pored with the former being either directly pored or pored via tubules which lead from the canal to the skin [11]. In some species, tubules may be ramified and form a highly complex network that provides information about close range water movements [13]. In contrast, non-pored canals are not exposed to external fluid movement and serve as tactile receptors while presumably decreasing the chance of particle interference in the canals [14].

The electroreceptive ampullae of Lorenzini, which are also embedded in the skin of elasmobranchs, provide complementary information about changes in close range electric fields [15]–[17]. Each ampullary pore connects to an individual ampulla by a single subcutaneous canal [18]–[19]. Sensory cells, which are located within the ampullary bulbs, analyse the voltage gradient between the internal environment of the ampullary bulb and the external environment surrounding the bulb [20]. In elasmobranchs, ampullae are often grouped into clusters or capsules according to their innervation [21].

Elasmobranchs typically possess between 500 and 1500 ampullary pores and the number of ampullae is positively correlated with electrosensory resolution [22]–[23]. While canal lengths increase with body size, the number of ampullae remains consistent ontogenetically [24]–[25]. Passive and structural electric properties dictate that longer canals are more sensitive to weak electric fields [26]. Thus, as elasmobranchs mature, their electroreceptive resolution decreases, while their sensitivity to weak electric fields increases [24].

The anatomical specialisations of the electrosensory system are related to each species' particular ecological niche. Correlations between electrosensory specialisations and foraging strategies have been well documented amongst elasmobranchs [10], [23]–[25], [27]–[31]. For example, sharks that inhabit the clear waters of the photic zone in the open ocean are largely considered visual predators that rely little on electroreception [32]. The blue shark, *Prionace glauca*, for instance, has one of the lowest pore counts recorded [33]. Turbid coastal environments, on the other hand, render visual prey localisation less reliable. Unsurprisingly, the coastal pelagic sharks *Carcharhinus plumbeus* and *Carcharhinus obscurus* possess more abundant pores [33]. In benthic elasmobranchs there are significant variations in ampullary arrangement, but generally pore numbers are increased ventrally to facilitate the detection of benthic prey [32]. However, benthic ambush predators such as *Orectolobus* sp. are almost devoid of ventral pores [30]. Their high concentration of dorsal pores is related to an overhead prey detection strategy [30].

Here, we examine the arrangement of the ampullary and lateral line systems of the epaulette shark *Hemiscyllium ocellatum* Bonnaterre 1788. These small, cryptic and benthic sharks inhabit coral reef flats of New Guinea and the Great Barrier Reef of Northeastern Australia [34]. On falling tide, epaulette sharks scavenge across the reef flat for benthic prey, such as small teleosts, polychaetes and crustaceans, which they consume using suction [34]–[35]. When searching for food, epaulette sharks move their heads laterally while swimming close to the substrate [35]. Upon prey detection, a shark arches its body thereby vertically lifting its tail and providing momentum to thrust its anterior end, up to the level of the first gill slit, into the sand [35]. Given their tendency to bury their heads in the sand, we hypothesise that the lateral line canals of the snout and ventral plane will not be pored. Although the development of their ampullary system is unknown, epaulette sharks are believed to use electroreception during foraging [35]. We thus predict that the ampullary system will be well developed with pores concentrated anteriorly and ventrally.

## Materials and Methods

### Ethics statement

Ethical approval was obtained from the James Cook University Animal Ethics Committee (Permit Number A1756).

### Study species

Mature specimens of *H. ocellatum* (n = 4, 2 females and 2 males, ranging in total length from 65.0 to 84.0 cm) were euthanized with a lethal dose of MS-222 (tricaine methane sulfonate; 1∶2000). Total length, fork length, sex and maturity were recorded and specimens were severed in the transverse plane behind the pelvic fins. Specimens were fixed in 10% neutrally buffered formalin for seven days and subsequently washed and transferred to a solution of 70% ethanol for storage.

### Dissections

**Figure 1:**
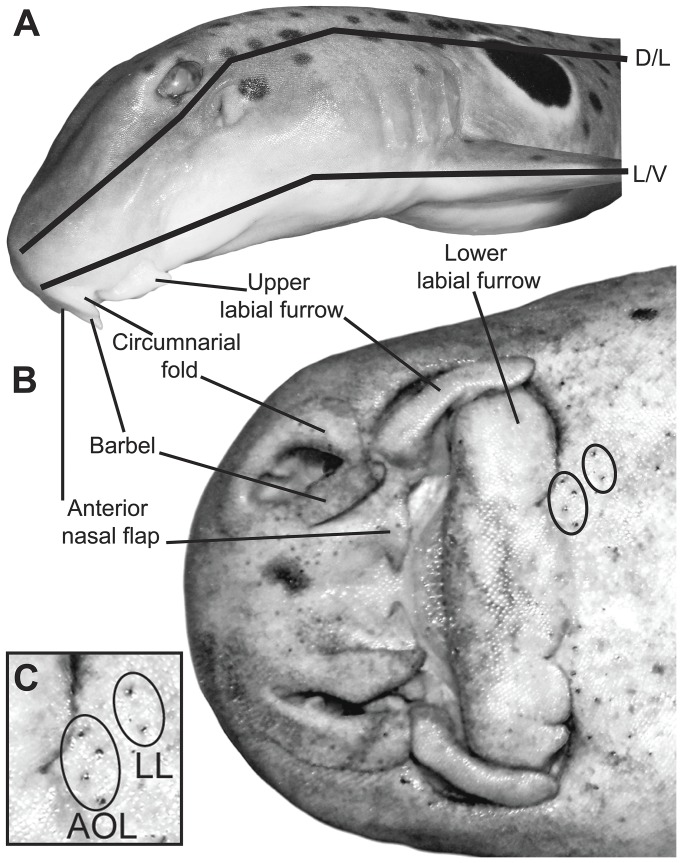
Study species: *Hemiscyllium ocellatum*. **A**) View of the head of *Hemiscyllium ocellatum* divided into dorsal (D), lateral (L), and ventral (V) planes. **B**) Ventral view of the head of *H. ocellatum* showing mouthparts specialised for benthic suction-feeding. **C**) The close physical association between electroreceptive (AOL) and mechanoreceptive (LL) pores in the skin.

The unique head morphology of the epaulette shark demanded partitioning into three planes: dorsal, lateral and ventral (Figure 1A). The relative positions of morphological features such as eyes, specialised mouth parts and fins were used to identify pore locations (Figure 1B). Externally, ampullary and lateral line pores are physically closely associated and difficult to distinguish (Figure 1C). Ampullary and lateral line pores were distinguished from each other by viewing their canals following the method of Wueringer and Tibbetts [29]. Prior to dissections, methylene blue (1%) was applied to the specimen's skin. Samples were viewed with an Olympus SZ40 stereo microscope. Ampullary structures were investigated by tracing independent canals from somatic pores to their associated ampullary bulbs. The length of ampullary canals from pore to ampullary bulb was measured *in situ* to avoid stretching of canals. Neighbouring pores with associated ampullae located in the same cluster were classed as a pore field. Diagrams were drawn as pores were discovered. In addition, individual pores were marked directly on samples to avoid overlapping counts. Single ampullae were viewed under an Olympus BX40 light microscope and images taken with a Nikon S4000 camera. During dissection of the lateral line canal system the presence of pored and non-pored areas was noted. Anatomical features are described according to the terminology of Chu and Wen [36] and Garman [37].

### Data processing

Representative diagrams were developed using Adobe Illustrator CS4 (www.adobe.com). Data were analysed using Statistics Plus 8.0. A Kruskal-Wallis rank sum test was used to determine if there was a significant difference between the number of ampullary pores associated with each ampullary cluster or each pore field. Similarly, differences in pore counts between dorsal, lateral and ventral planes were investigated. Variations in male and female pore counts were examined using the Wilcoxon rank sum test. A paired t-test was used to test for differences in pore abundance between left and right body-halves. Spearman's rank correlation examined a potential association between specimen length and total pore count.

## Results

The electroreceptive ampullary system and the mechanoreceptive lateral line canals are well developed in the cephalic region in *H. ocellatum* and both systems extend caudally.

### Lateral line canals

**Figure 2:**
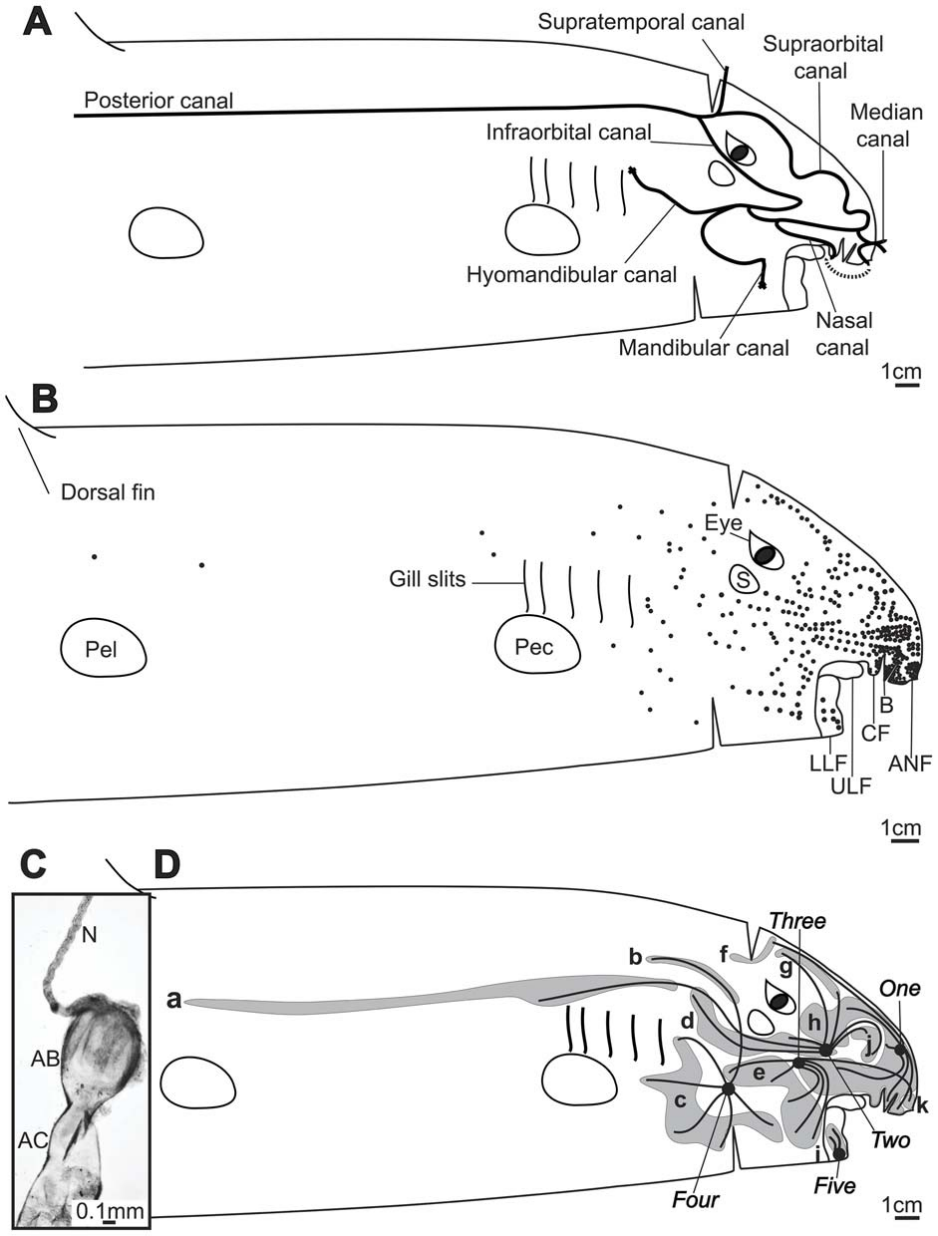
Arrangement of mechanosensory and electrosensory structures in *Hemiscyllium ocellatum*. **A**) Distribution of the mechanosensory lateral line canal. **B**) Distribution of electrosensory ampullary pores. Features designated; anterior nasal flap (ANF); barbel (B); circumnarial fold (CF); upper labial furrow (ULF); lower labial furrow (LLF); pectoral fin base (PEC); pelvic fin base (PEL); dorsal fin base (DOR); spiracle (S). **C**) Photomicrograph of an ampulla from cluster *three*, showing its associated ampullary canal (AC), bulb (AB) and nerve (N). **D**) Arrangement of ampullary pore fields (*a–k*) and ampullary clusters (*one–five*).

The lateral line canals of *H. ocellatum* form an evenly distributed network on all planes of the head (Figure 2A). All canals are open to the environment via regularly spaced pores. The posterior canal extends caudo-rostrally along the body axis. Posterior to the eye, it splits into three main paths; the supratemporal canal, the infraorbital canal and the supraorbital canal. The supratemporal canal connects across the midline caudally of the eyes. The infraorbital canal runs anteriorly between the eye and the spiracle until it splits into the mandibular canal and the hyomandibular canal. The mandibular canal draws ventrally where it arches towards the lower labial furrow and terminates in a single terminal pore. The hyomandibular canal curves towards the first gill slit where it ends in a single terminal pore. The third main path, the supraorbital canal, continues parallel to the median axis before drawing to the ventral plane of the snout where the median canal connects it across the midline of the snout. From there, the supraorbital canal bends posteriorly to connect to the nasal canal (posteriorly of the nostrils) and the infraorbital canal. The nasal canal runs along the posterior side of the nostril where it descends between muscle layers to form the only non-pored stretch of the lateral line canal. It further ascends to the cutis in the anterior nasal flap where it joins with the median canal.

### Ampullae of Lorenzini

*H. ocellatum* possess a total of 493 to 766 ampullae (mean ± sd dev. 646.25±132.29; Figure 2B, Table 1). Pores are concentrated on the snout and ventrally around the mouth (Figure 2B). Pores are also densely concentrated on the anterior nasal flap, barbel, circumnarial fold and lower labial furrow. Although pores extend posteriorly to the gills, pore density is reduced posterior to the eye. From the first gill slit, a line of 6±0.93 pores extends to the anterior juncture of the dorsal fin.

**Table 1:** Summary of the mean number of ampullary pores in *H. ocellatum*.

Pore field	Mean pore count ± sd	Cluster	Location
*a*	6.00±0.93	*Two*	Dorsal-Lateral
*b*	5.12±1.36	*Four*	Dorsal
*c*	15.50±1.93	*Four*	Ventral-Lateral
*d*	25.63±5.95	*Two*	Lateral-Dorsal
*e*	28.75±9.11	*Three*	Ventral-Lateral
*f*	2.57±0.79	*One*	Dorsal
*g*	15.38±2.13	*Two*	Dorsal
*h*	199.13±39.15	*One*	Ventral-Lateral-Dorsal
*i*	7.17±5.19	*Five*	Ventral
*j*	10.63±4.41	*Two*	Dorsal-Lateral
*k*	25.00±10.39	*Three*	Ventral
**Total**	323.13±65.44		

Pore counts are presented per pore field on one body half, according to their affiliation and location. Pores were counted in n = 3–8 pore fields each.

The ampullary structures of *H. ocellatum* form a complex network of long and short canals that link somatic pores and ampullary bulbs (Figure 2C). Ampullary bulbs are located in five distinct, bilaterally-paired clusters (Figure 2D). Although connective tissue is found between bulbs, there is no distinct capsule enveloping each cluster. The clusters are arranged in close physical proximity to the lateral line canals. Clusters *two* and *three* are separated only by loops of the nasal canal and the supraorbital canal along the frontal plane. Clusters *one* and *four* are also nestled alongside lateral line canals: anteriorly on the snout and ventrally prior to the gills, respectively. Eleven pore fields (*a–k*) are divided between the five clusters (Figure 2D). Most clusters receive input from multiple fields around the head (Table 1).

In *H. ocellatum*, ampullary canal lengths vary both between and within both pore fields and ampullary clusters (Figure 2D; Table 2). Across the four specimens, canals range in length from 2.71–244.37 mm. Cluster *two* and cluster *one* show the highest variation in canal lengths, while clusters *three* and *five* show the least variation.

**Table 2:** Summary of the length of ampullary canals in *H. ocellatum*.

Pore field	*a*	*b*	*c*	*d*	*e*
**Specimen 1**	21.3±8.9	4.9±0.8	3.5±1.5	4.6±2.6	2.9±1.0
**Specimen 2**	21.8±6.0	3.2±0.4	3.1±1.1	4.9±2.5	3.1±1.1
**Specimen 3**	17.3±5.9	5.0±1.3	2.9±1.4	5.3±2.6	3.0±0.9
**Specimen 4**	23.7±6.8	4.9±2.2	3.2±1.6	5.2±2.8	3.3±1.2

Pore field	*f*	*g*	*h*	*I*	*j*	*k*
**Specimen 1**	7.2±0.6	4.3±0.8	3.9±1.9	0.9±0.7	3.5±0.8	7.2±0.6
**Specimen 2**	6.8±1.5	4.2±1.0	3.2±1.3	1.1±0.6	2.6±0.8	6.8±1.5
**Specimen 3**	6.0±0.5	3.9±0.7	3.7±2.4	0.9±0.4	3.4±0.8	6.0±0.5
**Specimen 4**	7.8±1.4	4.4±0.9	3.8±1.3	0.8±0.5	2.6±0.4	7.8±1.4

Canal lengths are presented as a percentage of total body length (mean and standard deviation) per pore field. Total body lengths are as follows; specimen 1: 84.0 cm, specimen 2: 75.7 cm, specimen 3: 82.8 cm, specimen 4: 65.0 cm. Calculations are based on measurements from each pore field of the left lateral half of a specimen (n = 4).

Statistical comparison of the distribution of ampullary structures reveals that cluster *one* contains the most ampullae (Kruskal-Wallis rank sum test, X^2^ (4) = 32.21, P<0.01). Correspondingly, pore field *h* contains significantly more pores than any other pore field (Kruskal-Wallis rank sum test, X^2^ (10) = 68.84, P<0.01). Comparison of pore counts of the dorsal, ventral and lateral planes indicates that there is a significantly higher abundance of pores ventrally (Kruskal-Wallis rank sum test, X^2^ (2) = 15.38, P<0.001). However, there is no difference in pore counts between male and female specimens (Wilcoxon rank sum test, W (2, 2) = 6, P = 0.67). Specimens were analysed for symmetry between the left and right halves of the head and, although no specimen is perfectly symmetrical, the total number of pores on the left and right sides of the same do not differ significantly (Paired t-test, t (3) = 0.81, P = 0.48). There is no association between mean specimen pore count and specimen length (Spearman's rank correlation, r_s_ = −0.8, z (2, 4) = −1.56, P = 0.119).

## Discussion

The present study provides a detailed description of the anatomical specialisations of the electroreceptive and mechanoreceptive systems in the benthic epaulette shark *H. ocellatum*. Considering the available information on the ecology of this species, we propose functional aspects for the described morphological features. In addition, the unique distribution of ampullary pores on the body of the shark is discussed.

### Mechanoreception

We tested the hypothesis that cephalic lateral line canals of *H. ocellatum* would be non-pored. The hypothesis was derived from the mechanotactile hypothesis of Maruska and Tricas [38], which proposes that the non-pored canals in benthic batoids (skates and rays) facilitate benthic prey localisation by detecting tactile stimuli from infaunal organisms. These canals detect the velocity of skin movements generated when an external source depresses the skin above the canal [14]. Additionally, non-pored canals provide physical protection from intrusive particles, which could influence hydrodynamic flow. In this respect, they are thought to be necessary adaptations for benthic foragers.

Even though non-pored canals have been described in several benthic batoids [11], [14], [29], [39]–[42], only Maruska [11] described non-pored canals on the ventral side of a shark (bonnethead shark *Sphyrna tiburo*). Moreover, Chu and Wen [36] do not discriminate between directly pored and non-pored canals in their drawings. It thus remains unclear whether the mechanotactile hypothesis [38] also applies to benthic sharks. As the entire cephalic lateral line canal system of *H. ocellatum* is continuously pored, further comparative work is needed to identify the significance of these directly pored lateral line canals in the detection of benthic prey.

### Electroreception

The total number of ampullary pores in epaulette sharks appears to be low for a species that is thought to depend on electroreception during foraging. As epaulette sharks feed on benthic prey, they are unlikely to use vision during the final strike of prey capture when their heads are buried in the sand. However, epaulette sharks might not require a high electroreceptive resolution during the final stage of prey capture as these sharks are indiscriminate suction feeders [34]–[35]. In comparison, orectolobid wobbegong sharks, which are also indiscriminate suction feeders, also possess disproportionately low pore counts considering their likely dependence on electroreception [30]. This phenomenon can be explained by the fact that suction-feeding enhances a shark's strike radius and thereby relaxes the need for strike accuracy [43]–[44].

Further adaptations related to the direction of the feeding strike, and thus the location of the prey, are apparent within the electroreceptive systems of these suction-feeding sharks. In wobbegong sharks the highest densities of ampullary pores are located dorsally of the mouth, thereby enabling the detection and capture of prey passing overhead [30]. Epaulette sharks, on the other hand, possess high pore densities anteriorly and ventrally of the mouth. This pore arrangement should enhance its ability to scan large areas of substrate for prey, given that pores are widely spaced with canals radiating in all directions. As ampullae experience a maximum voltage gradient when the electric current is parallel to the canal axis [16], a localised prey electric field would provide highly differentiated input across all ventral electroreceptors. Once alerted, the shark's high concentration of anterior pores, particularly that of pore field *k*, could allow it to direct its otherwise indiscriminate suction-feeding strategy [44].

This study provides the first detailed description of ampullary structures on the body of a shark. Previously, it has been widely assumed that ampullary pores are restricted to the head of sharks [19], [32], [45]–[46]. In *H. ocellatum*, some pores of pore field *a* are located close to the pelvic fins and have canals which extend posteriorly over 29% of the total body length. When foraging and burying their anterior end in the substrate epaulette sharks may be particularly vulnerable to predation. The long posterior canals could alert the animal to weak electric fields of approaching predators and thereby facilitate a fast escape response. Alternatively, epaulette sharks may respond by performing a freeze response in the presence of a large predator. Embryonic skates in their egg capsules have been observed to respond in such a manner when presented with a large external electric field [47]. In this case, temporary cessation of ventilation rendered embryos less likely to be located by predators. The function of the unique pores of pore field *a* remains to be confirmed as does the shark's response to their stimulation.

The distribution of pore fields remains comparable amongst closely related elasmobranch taxa [24]. The extended canals of pore field *a* may be a common adaptation amongst hemiscyllid sharks that feed on benthic prey. The hemiscyllid shark *Chilioscyllium plagiosum* possesses pore fields comparable to those of *H. ocellatum*, including pore field *a* [36]. However, in *H. ocellatum* pore field *a* extends to the level of the pelvic fins, while in *C. plagiosum* the most posterior pores are positioned above the pectoral fins.

Comparison of the electroreceptive system of *H. ocellatum* and *C. plagiosum* with species studied by Ewart [48] allows speculation on the innervation of the clusters identified in *H. ocellatum*. We propose that cluster *one* represents the supraorbital cluster; cluster *two* represents the inner buccal cluster; cluster *three* represents the outer buccal cluster; cluster *four* represents the hyoidean cluster and cluster *five* represents the mandibular cluster.

### Conclusions

Both the electroreceptive and mechanoreceptive sensory systems are well developed in epaulette sharks. Continuously pored lateral line canals are distributed over the head and extend onto the body. The morphology of the ampullary system of epaulette sharks is concordant with the assumption that these animals rely on electroreception during foraging. However, low total ampullary pore counts may be accounted for by their indiscriminate suction-feeding strategy. As epaulette sharks inhabit clear, shallow and well-lit waters of reef flats and tide pools, they are thought to depend on vision during navigation of the complex reef topography [49]. Behavioural experiments are needed to confirm the role of both electroreception and mechanoreception in prey detection and predator avoidance. Finally, it is emphasised that electroreceptive structures are not limited to the cephalic region in sharks as previously widely assumed.
